# Food Environments around American Indian Reservations: A Mixed Methods Study

**DOI:** 10.1371/journal.pone.0161132

**Published:** 2016-08-25

**Authors:** Gwen M. Chodur, Ye Shen, Stephen Kodish, Vanessa M. Oddo, Daniel A. Antiporta, Brittany Jock, Jessica C. Jones-Smith

**Affiliations:** 1 Center for Human Nutrition, Department of International Health, Johns Hopkins Bloomberg School of Public Health, Baltimore, Maryland, United States of America; 2 Department of Nutrition, Harvard T.H. Chan School of Public Health, Boston, Massachusetts, United States of America; 3 Global Obesity Prevention Center, Johns Hopkins Bloomberg School of Public Health, Baltimore, Maryland United States of America; 4 Department of Social and Behavioral Interventions, Johns Hopkins Bloomberg School of Public Health Baltimore, Maryland United States of America; University of Tennessee Health Science Center, UNITED STATES

## Abstract

**Objectives:**

To describe the food environments experienced by American Indians living on tribal lands in California.

**Methods:**

Geocoded statewide food business data were used to define and categorize existing food vendors into healthy, unhealthy, and intermediate composite categories. Distance to and density of each of the composite food vendor categories for tribal lands and nontribal lands were compared using multivariate linear regression. Quantitative results were concurrently triangulated with qualitative data from in-depth interviews with tribal members (n = 24).

**Results:**

After adjusting for census tract-level urbanicity and per capita income, results indicate there were significantly fewer healthy food outlets per square mile for tribal areas compared to non-tribal areas. Density of unhealthy outlets was not significantly different for tribal versus non-tribal areas. Tribal members perceived their food environment negatively and reported barriers to the acquisition of healthy food.

**Conclusions:**

Urbanicity and per capita income do not completely account for disparities in food environments among American Indians tribal lands compared to nontribal lands. This disparity in access to healthy food may present a barrier to acting on the intention to consume healthy food.

## Introduction

American Indian/Alaskan native (henceforth American Indian) are disproportionately burdened by non-communicable diseases, such as obesity and diabetes, when compared to white Americans [1–3]. The estimated prevalence of obesity is 40% of American Indian adults compared to 28% among whites. Similarly, 20% of American Indians are estimated to have diabetes versus 12% of whites [4]. Environmental characteristics of tribal communities, including access to healthy and unhealthy food, may contribute to these health disparities [5, 6]. Nationwide studies have found that neighborhood availability of healthy and unhealthy food sources vary systematically by neighborhood race/ethnic composition and median household income in the U.S. Low-income neighborhoods as well as neighborhoods where the majority of the population is Black or Hispanic are less likely to have a supermarket [7], but more likely to have a fast food vendor or other less healthy food options compared to higher income neighborhoods or those where the majority of the population is not Black [5, 8, 9].

Few studies have examined whether similar disparities in neighborhood food environments exist for American Indian communities. Some evidence from studies of American Indian communities in Arizona, New Mexico, and Washington suggest that those living on tribal lands may be more likely to experience barriers, such as high cost of food and transportation challenges, in the acquisition of healthy food [10–12] in addition to low food security [10, 11]. However, a large proportion of tribal communities exist outside of these three states and may have differing environmental characteristics. California is home to 13.9% of all American Indians nationally [13] with 109 federally-recognized tribes [14]. The large proportion of tribal communities represented in this state, coupled with the evidence linking neighborhood environments and health outcomes, highlights a need for research that describes current environments in order to positively shape intervention and policy development [15–18].

The primary aim of this study was to explore and describe the food environments on tribal lands as compared to non-tribal lands, using qualitative and quantitative data. We hypothesized that American Indians living in these settings face heightened barriers to the attainment of healthy food, including unique challenges related to geographic and economic food access.

## Methods

### Study Design

We employed a concurrent triangulation design that allowed for comparison of qualitative and quantitative food environment findings [19]. This mixed methods study used in-depth interviews with tribal-affiliated members about their food environments in order to triangulate quantitative data comparing food store availability in both tribal and non-tribal communities. Participants were tribal members of one of the 109 tribes in CA, who occupy the same 94 tribal lands in California that are investigated with the quantitative food environment data. Residence on tribal land was not criteria for inclusion; however, participants who did not live on tribal lands were questioned about the food environment surrounding tribal lands. Nearly all members we interviewed lived on or near tribal lands and, in that way, they are connected to the quantitative sample.

Below we first describe our quantitative methods and then describe the qualitative methods.

### Quantitative Procedures

The quantitative analysis aimed to describe the food environment of California tribal lands by comparing indicators of food availability for communities with tribal lands to those without tribal lands. Quantitative and geospatial data were derived from U.S. Government sources, and statewide food business data were obtained from InfoUSA for California in 2013. We limited our sample to census tracts within counties containing at least one tribal land (n = 29 of 58 counties in California).

#### Dependent Variable

Our dependent variables included the density of and distance to healthy, unhealthy and intermediate-healthy food outlet composites. Variable creation was a multistep process briefly described below and detailed in S1 Text.

#### Food Environment Variable Construction

We classified all individual food vendors into food business categories based on commonly used definitions similar to those used by Rundle et al[20]. The primary and secondary Standard Industrial Classification (SIC) codes associated with each food business in the InfoUSA dataset provided classifiable information about the venue. Thus, these SIC codes were used to categorize all food establishments as either a supermarket, superstore, produce market, sit-down restaurant, carryout restaurant, fast food restaurant, convenience store or gas station, healthy specialty store, mixed (healthy and unhealthy) specialty store, or an unhealthy specialty store (See S1 Text for detailed procedures of the classification, and S1 Table for corresponding categories of the specific SIC codes). Additional special modification was made to capture top fast food chains to the fast food restaurant category by text matching (See S2 Text for the list of included fast food chains).

For the analyses, the 14 categories were further collapsed into 7 categories: 1. Supermarkets, Superstores, and Produce markets; 2. Fast Food; 3. Carryout and Unhealthy Specialty Stores; 4. Mixed Specialty Stores; 5. Healthy Specialty Stores; 6. Sit-Down restaurants; 7. Convenience Stores and Gas Stations.

Finally, from these seven categories, we created three composite categories: 1) Healthy Outlets (1 & 5); 2) Intermediate Outlets (4 & 6); and 3) Unhealthy Outlets (2 & 3 & 7).

Afterwards, each food business was geocoded using ArcGIS for geospatial analyses. Density per square mile of healthy, unhealthy and intermediate food vendors was then calculated for each geography (described below). Additionally, the distance along road networks from each geography to the nearest food business in each category of the food vendors was calculated.

#### Independent variables

Our primary independent variables were binary variables indicating a geography as an American Indian tribal area. We used two slightly different geographies to define tribal areas in these analyses. For the *density* analyses of food vendors, tribal area was defined as U.S. census tract boundaries that encompassed the tribal land because actual tribal lands in California are typically very small and have very few businesses located on these lands. Therefore, to capture what we hypothesize to be the relevant food environment for American Indians living on tribal lands, the density analyses utilized the larger U.S. census tract boundaries that encompassed the tribal land to include the nearby food environment. Census tracts without tribal lands were used as the comparison (i.e. non-tribal areas). For the analyses of *distance* to the nearest food vendors, tribal area was defined based on the U.S. “tribal census tract”[21] and distance from the centroid of the tribal census tract to food vendors was calculated. Tribal census tracts include only the land area of federally recognized American Indian reservations or tribal lands. Distance from the centroid of census tracts without tribal lands was used as the comparison (i.e. distance for non-tribal areas). Henceforth, for readability, we refer to the comparisons of these specific geographies as tribal areas versus non-tribal areas. Census tracts eligible to be included in the distance analysis (n = 2,427) had 49 cases of missing information (per capita income, n = 28; urbanicity, n = 5; distance, n = 16) leaving 2,378 census tracts, 94 of which were tribal census tracts (tribal areas). On the other hand, 2,364 census tracts were eligible for the density analyses, but 12 were missing information about per capita income, leaving a total of 2,352 census tracts for the density analyses, 52 of which contained tribal lands.

Census tract polygon shapefiles (U.S. 2010 Census), tribal tract polygon shapefiles (U.S. 2010 Census), and road line shapefiles (U.S. TIGER) were used to determine the geographies of interest and to conduct all the geospatial analyses described above using ArcGIS.

#### Descriptive Characteristics and Covariates

Total population, land area, race/ethnic composition, population density, and household urbanicity at census tract-level were obtained from the 2010 U.S. Census. Five-year estimates (2009–2013) of per capita income and percent with a high school diploma were obtained from the American Community Survey. Per capita income was adjusted for inflation and presented in 2010 U.S. dollars.

#### Statistical Analysis

We used two-sided t-tests with unequal variance to compare the mean distance to the nearest food vendor and the density of food vendors under each of the 3 composite food vendor categories and 7 subcategories for tribal areas compared to non-tribal areas. To assess food environment differences after controlling for two key area-level characteristics that we hypothesized to be potentially influencing the food environment (urbanicity and per capita income), we used separate adjusted multivariable linear regression models. Specifically, we assessed the association between 1) tribal areas and distance to each of the 3 composite categories and 7 subcategories of food vendors and 2) tribal areas and the density of the 3 composite categories plus 7 subcategories of food vendors (See S1 Dataset for the per-tract level data). Models included mean-centered per capita income and mean-centered percent of the population living in urban areas as continuous variables. Alpha was set to 0.05. Statistical analyses were performed using Stata 13.1 (College Station, Texas).

### Qualitative Procedures

We conducted semi-structured, in-depth interviews with tribal members (*n* = 24). The original purpose of the interviews was to explore the impact of having and not having a casino on tribal member weight-related health, which has been reported elsewhere; however, the interview guide also included questions about the general environment around tribal lands, tribal culture and context, food acquisition, and perceptions of and experiences with the food environments, which were used for the purposes of this study. For example, the interview guides included questions such as: Can you please describe the community where you live to me?; Where does your family get most of the food you have in the house?; Please describe a typical trip to get groceries.; What do you see as the most important things in your community that influence your weight? Your children’s weight?; What are the major things you consider when deciding what you and your family eat?

#### Sampling and recruitment

Tribal members (*n* = 24) were purposively sampled from 109 federally-recognized California tribes (15) by study staff at health clinics and conferences, as well as through word-of-mouth referrals [22]. Specifically, flyers were sent to the all of the tribal health clinics in California and study staff contacted the tribal health clinics to request that the flyers be posted. Participants responding to a flyer were screened for eligibility over the telephone. Participants were eligible for an interview if they were at least 18 years of age, spoke English fluently, and were either a tribal member themselves or had a child who was a tribal member. Interviews were scheduled with eligible participants, the majority of which took place over the phone. We sought to interview participants who were information-rich: willing to talk at length and with great insights about themselves and their communities [23, 24]. By using stratified purposive sampling to obtain respondents based on geographic location throughout the state, tribal size, and affiliation with a casino, we aimed understand the perspectives of different types of tribal members [25]. Participants were sampled until data saturation was reached among key thematic areas of inquiry [26, 27].

#### Data Collection

Interviews were conducted with tribal members between May 2014 and April 2015 using semi-structured guides that included questions about community characteristics, tribal culture and context, perceptions of the food environment surrounding tribal areas, and food shopping and eating patterns. Interviews were conducted in-person or via telephone, ranged between 40–60 minutes, and were digitally recorded. All interviews were conducted by the principal investigator or graduate students trained in qualitative interviewing. Interviews were conducted until data saturation was reached among key themes (27–29).

#### Qualitative Data Analysis

During data collection, study staff held weekly meetings to discuss emergent themes from interviews. This team-based approach allowed for flexibility and iteration [24], with methodological decisions related to instrument revision and sampling strategy being made throughout the study period.

The textual analysis proceeded in a step-wise process. First, digital recordings were transcribed with identifying details redacted. Three team members developed a codebook with descriptive categories based on interview guide content and study objectives [28]. Systematic definitions and instructions were created to ensure consistent use of the codebook among multiple coders [29]. Second, three transcripts were inductively coded using Dedoose software to identify emergent themes that had not been initially identified and create a coding frame with 18 codes. Third, following suggested guidelines for establishing inter-coder reliability among teams of individuals [30, 31] one team member initially coded one-quarter of the data set (6 transcripts) and the two other team members blindly re-coded the same transcripts in order to estimate rater agreement across many codes using Kappa scores [32]. This effort yielded scores of 0.71 and 0.78, suggesting “good” reliability based on established cutoffs [33]. The team then coded 50% more of the data set, making codebook modifications and re-coding text when necessary to improve reliability [34]. A second reliability test then generated scores of 0.84 and 0.83, considered “excellent” reliability [33]. The team coded the remainder of the data set before comparing the qualitative findings to the quantitative results for interpretation.

#### Ethics Statement

Participants, both those in person and those interviewed by telephone, were read a consent statement and given the opportunity to ask questions. All participants provided oral consent which was logged on a tracking sheet prior to the initiation of the interview. The research proposal and oral consent procedure were reviewed and approved by the Institutional Review Board at the Johns Hopkins Bloomberg School of Public Health and the California Rural Indian Health Board.

### Integrating the Quantitative and Qualitative Data

Analysis of quantitative and qualitative data were conducted separately and then compared. Specifically, results from the qualitative and quantitative work were presented at research team meetings including the qualitative and quantitative analysts. The group then discussed in-depth how the main quantitative findings compared with themes that had emerged from the qualitative work. For example, when the quantitative analyst finished the unadjusted analyses of whether distance to healthy and unhealthy food vendors was different for tribal lands compared to non-tribal lands, the team discussed whether the emergent themes from the qualitative data analysis were consistent with this set of findings or not. When we found some unexpected results in the quantitative analysis after adjusting for area level factors, we consulted the qualitative data analysts to see whether there were any less salient points of view that would have been consistent with the quantitative findings. We identified places where the quantitative and qualitative results were consistent and places in which the results diverged and we report both below.

## Results

### Overview

As mentioned above, the analysis of quantitative and qualitative data were conducted separately and then compared. After describing the sample characteristics for both quantitative and qualitative data, we present the quantitative findings first, then followed by the qualitative findings that were relevant to each of the main quantitative outcomes, namely density and distance. There were three themes that emerged in the qualitative data that were populated with content relevant to describing the food retailer mix surrounding tribal areas. These themes were labeled challenges in acquiring food,” “eating out” and “negative perceptions of the food environment”. Similarly, the themes labeled “reasons for using primary food source” and “challenges in acquiring food.” were populated with content that was about the distance to food venues.

### Sample Characteristics

Demographic and socioeconomic characteristics of tribal and non-tribal areas included in the quantitative analysis are displayed in Table 1. Only 30% of households in tribal areas are located in urban areas, compared to 89% in non-tribal areas.

**Table 1:** Socio-Economic and Demographic Characteristics for All Census Tracts, Census Tracts with and without Tribal Land, and Tribal Census Tracts.

	All Census Tracts^a^ (*n* = 8024)		Census Tracts with Tribal Land^b^ (*n* = 52)		Census Tracts without Tribal Land^c^ (*n* = 2300)		Tribal Census Tracts^d^ (*n* = 94)	
	Mean	SD	Mean	SD	Mean	SD	Mean	SD
**Socio-demographic data**								
Population density (per square mile)	8,369	9,490	43	71	4,553	4,529	506	664
Urban Households %	94^d^	21	21	28	90	26	30	42
**Race/ Ethnicity**								
White %	59	20	79	9.2	66	17	0	0
Black %	6.2	9.6	1.1	1.9	4.8	5.5	0	0
Asian %	13	15	1.2	1.1	6.7	7.9	3.1	8.3
AIAN %	1.0	1.6	6.8	6.5	1.3	2.3	53	29
Latino or Hispanic %	36	26	18	13	37	24	18	21
**Socio-Economic Indicators**								
Average Per Capita Income($)^e^	30,320^f^	17,875	25,783	6,799	26,449	12,857	21,955	16,136
High School Diploma %	80	17	86	6.9	81	15	77	16
Median Household Income($)^e^	66,049^f^	31,058	50,849	16,630	59,311^f^	25,113	43,941^f^	20,454
Area (square miles)	1.8	15	61	103	2.9	20	16	51

*Note*. SD, Standard Deviation; AIAN, American Indians and Alaska Natives

^a^ All census tracts in California

^b^ Census tracts that contain any tribal lands

^c^ Census tracts without tribal lands in counties with tribal lands

^d^ Tribal Census Tracts as defined in US 2010 Census and include primarily the land area of federally recognized American Indian reservations or off-reservation tribal lands

^e^ Adjusted for inflation to 2010 dollar

^f^ Sample sizes differed slightly due to missing data on some characteristics. Sample size of all census tracts for average per capita income = 8010.Sample size of all census tracts for median household income = 7969. Sample size of census tracts without triabl lands for median household income = 2293. Sample size of tribal census tracts for median household income = 87.

Approximately 53% of the population in the tribal areas identified as American Indian, compared to 1% in non-tribal areas. Average per capita income was substantially lower for tribal areas compared to non-tribal areas (Table 1). Demographics of the qualitative sample are presented in Table 2. Participants were primarily female and more than half were from tribes with more than 1000 members.

**Table 2:** Demographic Characteristics of Participants Providing Qualitative Interviews.

	Number	Percent of Sample
**Gender** (n = 24)		
Male	4	17%
Female	20	83%
**Geographic Location** (n = 24)		
Northern California	15	63%
Central California	2	8%
Southern California	7	29%
**Tribal Population** (n = 23)		
<500	7	30%
500–1000	5	22%
>1000	11	48%

### Density per square mile of food vendors comparing tribal to non-tribal areas

Bivariate analyses exploring the density of food outlets per square mile indicated that for all composite categories (healthy, unhealthy and intermediate) and all subcategories of food vendors, there were significantly fewer food vendors for tribal areas compared to non-tribal areas (Table 3). After adjusting for tract-level percentage of household in urban areas and per capita income, results indicate there were significantly fewer healthy food outlets per square mile for tribal areas compared to non-tribal areas (*b* = -0.063; 95% CI: -0.11, -0.015) (Table 4). Density of unhealthy outlets (*b* = -0.20; 95% CI: -0.47, 0.07) and intermediate outlets (*b* = -0.14; 95% CI: -0.42, 0.14) were not significantly different for tribal versus non-tribal areas (Table 4).

**Table 3:** Bivariate Results^a^ for Density of and Distance to Food Outlets between Tribal Areas^b^ and Non-Tribal Areas^c^.

Mean	Healthy Outlet Composite	Supermarkets, Superstores, & Produce Markets	Healthy Specialty Stores	Intermediate Outlet Composite	Mixed Specialty Stores	Sit-down Restaurants	Unhealthy OutletComposite	Fast Food	Carryout & Unhealthy Specialty Stores	Convenience Stores & Gas Stations
N	15,303	12,037	3,280	47,419	1,604	46,015	44,609	16,812	18,488	9,415
**Density** ^d^										
Census Tracts with Tribal Land	0.0020	0.0014	0.00037	0.0031	0.000098	0.0030	0.0035	0.0011	0.0011	0.00078
Census Tracts without Tribal Lands	0.26	0.20	0.036	1.2	0.018	1.1	1.3	0.44	0.44	0.21
Difference ^e^	0.26	0.20	0.036	1.2	0.018	1.1	1.3	0.44	0.44	0.21
p value	<0.001	<0.001	<0.001	<0.001	<0.001	<0.001	<0.001	<0.001	<0.001	<0.001
**Distance** ^f^										
Tribal Census Tracts	6.0	6.0	20	6.3	28	6.4	8.5	12	11	11
Census Tracts without Tribal Lands	1.7	1.8	3.4	1.6	5.5	1.6	1.7	2.1	2.1	2.0
Difference^e^	-4.2	-4.2	-17	-4.7	-22	-4.8	-6.8	-9.9	-8.7	-8.5
p value	<0.001	<0.001	<0.001	<0.001	<0.001	<0.001	<0.001	<0.001	<0.001	<0.001

^a^ T-test with unequal variance. All numbers were rounded to two significant digits.

^b^ In the distance analyses, tribal areas refer to “tribal census tracts” as defined by the US Census (n = 94) including primarily the land area of federally recognized American Indian reservations or off-reservation tribal lands. In the density analyses, tribal areas refer to census tracts that contain tribal lands (n = 52)

^c^ Non-tribal areas refer to census tracts without tribal lands (n = 2284 for distance, n = 2300 for density) in counties with tribal lands

^d^ Density of food outlets per square mile was obtained for each census tract using the kernel density estimation

^e^ difference = nontribal–tribal; differences are the same as the census tract without tribal lands due to rounding.

^f^ Distance is calculated as the shortest distance along the road network from the tribal census tract centroid to the closest food outlet of the respective category.

**Table 4:** Multivariate Linear Regression results^a^ for density of and distance to food outlets for tribal areas^b^ compared to non-tribal areas^c^.

	Healthy Outlets Composite	Supermarkets, Superstores & Produce Markets	Healthy Specialty Stores	Unhealthy Outlets Composite	Fast Food	Carry-out Restaurants & Unhealthy Specialty Stores	Convenience Stores & Gas Stations	Intermediate Outlets Composite	Sit-down Restaurants	Mixed-Specialty Stores
**Density**^d^										
β (95%Confidence Interval)										
Tribal Areas^b^	-0.063 (0.11, -0.015)*	-0.051 (-0.088, -0.013)*	-0.011(-0.017, -0.0055)*	-0.20 (-0.47, 0.073)	-0.082 (-0.17, 0.003)+	-0.066 (-0.16, 0.031)	-0.053 (-0.091, -0.015)*	-0.14 (-0.42, 0.14)	-0.14 (-0.40, 0.13)	-0.0057 (-0.0088, -0.0027)*
Constant (Non-tribal Areas^c^)	0.25 (0.25, 0.26)*	0.20 (0.19, 0.20)*	0.034 (0.034, 0.035)*	1.2 (1.2, 1.3)*	0.42 (0.41, 0.43)*	0.41 (0.39, 0.42)*	0.21 (0.20, 0.21)*	1.1 (1.0, 1.1)*	1.0 (1.0, 1.1)*	0.017 (0.017, 0.018)*
**Distance**^e^										
β (95%Confidence Interval)										
Tribal Areas^b^	-0.20 (-0.81, 0.42)	-0.22 (-0.83, 0.39)	7.3 (5.4, 9.2)*	1.7 (0.93, 2.5)*	3.1 (2.2, 4.0)*	2.0 (1.1, 2.9)*	2.5 (1.6, 3.3)*	0.51 (-0.095, 1.1)+	0.51 (-0.10, 1.1)	9.7 (7.3, 12.1)*
Constant (Non-tribal Areas^c^)	1.9 (1.8, 2.0)*	2.0 (1.9, 2.1)*	3.8 (3.5, 4.2)*	1.9 (1.8, 2.1)*	2.4 (2.2, 2.5)*	2.4 (2.3, 2.6)*	2.3 (2.1, 2.4)*	1.8 (1.7, 1.9)*	1.8 (1.7, 1.9)*	6.0 (5.6, 6.5)*

* p <0.*05 + p<0*.*1*

^a^ Regression model is adjusted for per capita income and percent of the population living in urban areas; distance regressions (n = 2378) and density regressions (n = 2352). All numbers were rounded to two significant digits

^b^ In the distance analyses, tribal areas refer to “tribal census tracts” as defined by the US Census including primarily the land area of federally recognized American Indian reservations or off-reservation tribal lands. In the density analyses, tribal areas refer to census tracts that contain tribal lands

^c^ Non-tribal areas refer to matched census tracts without tribal lands that belong to counties with tribal lands

^d^ Density of food outlets per square mile was obtained for each census tract using the kernel density estimation

^e^ Distance is calculated as the shortest distance along the road network from the tribal census tract centroid to the closest food outlet of the respective category

### Tribal members’ perception of local food environment options

Tribal members’ perception of their food environment was generally consistent with the aforementioned density findings which suggested significantly fewer healthy food vendors, yet no difference in unhealthy food vendors. Many participants in the qualitative interviews had negative perceptions of the quality of food available to them.

*“[B]ecause there's not really a whole lot of healthy choices*. *It's just like*, *fried food and deep fried food*,” (Mother of seven, Northern California). And, “*I think we need to have healthier*, *more choices around here*. *There’s really not that much*. *I see a lot of fast food and stuff like that”* (Mother of four, Northern California).

Many participants expressed that the lack of convenient healthy food options limited their ability to acquire and consume healthy food because of limitations in affordability and availability in spite of the desire to provide healthy food for their families.

Many participants responded that they faced limited options in where they could acquire groceries.

*“It’s the only grocery store here”* (Mother of two, Northern California). “*We have to drive into a small town that doesn’t really have any grocery stores*. *That’s been out of the way*. *The closest town is probably 5 miles away”* (Mother of five, Southern California).

The perceived availability of fast food and sit-down restaurants were the most divergent views from participants. While some reported there was no fast food available, others described the presence of multiple fast food chains.

“*We don’t have fast food*, *so that’s a big thing”* (Mother of three, Northern California). *“[T]here’s like Taco Bell*, *Burger King*, *Jack in the Box*, *McDonalds*, *and KFC in one round-a-round*, *so it’s like right here”* (Mother of four, Northern California).

### Distance to food vendors for tribal areas compared to non-tribal areas

In bivariate analyses, distance to each composite category (healthy, unhealthy and intermediate) and subcategory of food vendors was significantly further for tribal areas as compared to non-tribal areas (Table 3). In models adjusting for percent of the population living in urban areas and per capita income, distance estimates to healthy, unhealthy and intermediate food vendors yielded mixed results (Table 4). Adjusted distance to unhealthy food vendors was 1.7 miles (*b* = 1.7, 95% CI: 0.93, 2.4) longer in tribal areas as compared to non-tribal areas. However, distance to healthy food outlets (*b* = -0.20; 95% CI: -0.81, 0.42) and intermediate food outlets (*b* = 0.51; 95% CI: -0.10, 1.12) were not significantly different in tribal areas compared to non-tribal areas.

### Tribal members’ perceptions of distances to food vendors

The tribal members’ perspectives were more aligned with the unadjusted bivariate results, indicating a further distance traveled for tribal compared to non-tribal areas. When asked about a typical trip to the grocery store, many participants described challenges and stated that distance to the closest grocery store was an influential factor in choosing foods to purchase.

*“[A typical trip to buy groceries is] a lot of work*. *Driving 20 miles*, *which is a half an hour more because of the roads*. *First of all because five kids to go shopping with*, *and cost*, *the price of food*.*”* (Mother of five, Northern California).

*“[W]e don’t have a local grocery store*. *Well*, *we have a little gas station*, *but I have to round up all the kids*, *get them in the car*, *and drive 10 miles just to go get groceries*.” (Mother of three, Northern California).

Some participants reflected on how the distances to a supermarket affected their diet quality.

*“I think it affects the quality because going down the mountain to eat–you only buy enough for a week to keep it fresh*. *If I buy the vegetables*, *or a heavy amount of shopping I know that realistically I only have enough gas to go down there… So*, *vegetables I know a lot of them aren’t going to last 10 days*,*”* (Mother of five, Southern California).

## Discussion

California is home to 109 federally recognized tribes and 94 tribal areas [14]. Of these tribal areas, approximately half are Rancherias, which are smaller areas of land compared to reservations. These tribal lands are spread throughout the state and vary greatly in terms of geography. Using data covering the tribal lands in California, bivariate analysis of density showed that tribal areas have significantly fewer of both healthy and unhealthy food businesses. After adjusting for community-level urbanicity and average per capita income, we found that tribal areas still have significantly fewer healthy food vendors (supermarkets, superstores, produce markets, and healthy specialty stores) per square mile compared to non-tribal areas. However, no significant differences were found between tribal and non-tribal areas in the density of unhealthy food vendors (fast food, carry-out restaurants, unhealthy specialty stores, and convenience stores and gas stations). Bivariate analyses of distance indicated longer distances to all food vendors; however, adjusted analyses suggested that only distance to unhealthy healthy food vendors was significantly longer for tribal areas compared to non-tribal areas. Interviews with tribal members were largely consistent with the findings of a low density of healthy foods and with unadjusted distance findings, indicating longer distances to all food vendors.

### Density of healthy and unhealthy food vendors

In adjusted models, we found that tribal areas tended to have fewer healthy food vendors per square mile but no significant difference in unhealthy food vendors, suggesting that the disparity in food availability for tribal areas compared to non-tribal areas is not entirely due to the lower urbanicity or lower average per capita income. Although this is the only study to our knowledge to compare the food environments of tribal to non-tribal areas, our findings on food availability are congruent with other research that shows differential access to healthy and unhealthy food vendors according to neighborhood racial/ethnic composition. Specifically, Powell et al. report that neighborhoods with a higher proportion of Blacks are more likely to have fast food vendors and less likely to have supermarkets compared to neighborhoods with a high proportion of whites [7]. Previous research in other settings has also found lower availability of supermarkets in areas with relatively higher proportion of minority residents [35, 36]. We found no significant difference in the density of unhealthy food options, which is in contrast to the findings of Powell and Lamichhane, who found that neigborhoods with lower income or higher proportion of minority populations had higher availability of unhealthy food options [7, 35]. Our findings also differ from evidence suggesting lower density of unhealthy food in socioeconomically disadvantaged neighborhoods[37].

### Distance to healthy and unhealthy food vendors

After adjusting for tract-level urbanicity and per capita income, there was no difference in the distance to the nearest *healthy* food vendor for tribal areas compared to non-tribal areas. However, *unhealthy* vendors were significantly farther away for tribal areas compared to non-tribal areas. At the same time, a prevailing theme in many interviews was the challenges faced in acquiring food, including long travel distances and negative perceptions of the immediate food environment. These seemingly discrepant findings are likely explained by the fact that tribal members are responding to their actual environment rather than a regression-adjusted environment. Indeed, the bivariate analysis showed that distance to both healthy and unhealthy food vendors were significantly longer for tribal areas compared to nontribal areas, a reflection of the actual food environment experienced by American Indians living in tribal areas. The purpose of multivariate regression is to examine if additional disparities exist beyond what could be explained by urbanicity and socio-economic status, although people living in these areas experience the cumulative effects of these factors. In addition, it is possible that the qualitative findings captured other aspects of accessibility, such as increased price or decreased quality at the healthy vendors closest to tribal tracts rather than simply distance, as previous research has found higher costs for foods of high nutritional quality in rural, impoverished areas when compared to more wealthy urban areas [38]. The method used to calculate distance may not adequately account for travel time, which may be increased on rural or mountain roads in tribal areas and therefore contribute to a perceived burden associated with attempts to acquire healthy food.

Similar to disparities in access that have been documented in neighborhoods with a higher proportion of black or Hispanic residents, even after controlling for income, we had hypothesized that tribal areas would experience similar disparities in access even after controlling for general degree of urbanicity. We speculate that this is the case partially due to an even greater remoteness of American Indian tribal areas compared to rural areas in general stemming from the historical failures on the part of the US government to honor land treaties and a motivation to settle American Indians on lands that were as distant as possible from white settlers.[39]. These results are consistent with other research examining healthcare accessibility in American Indian communities which has found lower healthcare access in rural communities with high concentrations of American Indian populations compared to rural areas without American Indian populations [40]. As has been speculated for inner city predominantly black neighborhoods, there may be reluctance for businesses to locate in these places due to systematic racism [7]. Supermarkets, the primary source of healthy food, may be particularly sensitive to these dynamics, while smaller sources of unhealthy food such as convenience stores or fast food outlets may more readily locate in such areas [41].

Finally, tribal members perceived the immediate food environment negatively, providing unfavorable characterizations of the quality of food available to them. They reported that limited options available at food outlets, insufficient finances for “organic” foods, and the abundance of “junk foods” thwarted their ability to carry through on intentions to purchase and consume healthy foods. These other considerations help to contextualize the quantitative findings and reconcile differences between the quantitative and qualitative results, as distance is not the only consideration made when considering where to acquire food. The perceptions of having to travel a far distance to food vendors and of a generally unhealthy food environment among the tribal members surveyed are consistent with findings that increased distance to supermarkets was associated with decreases in perceived availability of healthy food in a rural context [42]. Our findings are also consistent with Rose’s economic model of food choice [43], which suggests negative perceptions of the food environment are held by populations who face high travel costs in the acquisition of food [44].

Limitations should be noted. First, we used commercial business data rather than verifying business locations in person to characterize the food environment. However, since our research covered large portions of California, this was the only option. This limitation also prevented in-store quantification of food availability; an implicit assumption of our analysis is that all stores within a category were homogenous in the quality of their food offerings, when heterogeneity between stores of the same category is likely. Additionally, the food environments described by participants in the qualitative sample do not necessarily reflect the food environments experienced by populations in all tribal areas in California and may not reflect the multitude of experiences of American Indians. Although we report on rich qualitative data, we did not systematically collect a larger array of sample characteristics, such as age, socioeconomic indicators, or participation in Food Distribution Program on Indian Reservations or other food assistance programs. We are therefore limited in our ability to characterize the sample according to those features or draw any conclusions about differences based on those characteristics. Additionally, as previously described, the geographic area of interest was different for the analyses that examined food vendor density and those that examined distance to nearest food vendor. Although this complicates the description and interpretation, we felt that the geographic unit used for each was the most relevant unit for each exposure. Finally, our qualitative sample does not include perceptions from non-tribal members or people living in non-tribal areas in California.

## Conclusion

This study enhances our understanding of the food environment surrounding tribal areas. In particular, our adjusted analyses suggest that the disparity in food availability for tribal areas compared to non-tribal areas in California is not entirely due to urbanicity and per capita income. Additionally, our qualitative analysis suggests that individuals living in tribal areas perceive significant barriers to the acquisition of healthy food. A better understanding the food environment surrounding tribal areas is critical given the potential role of those environments in the disproportionate burden of obesity and diabetes among American Indian populations. This research suggests that interventions to improve diet-related health outcomes in these communities should consider the structural barriers to access faced by many populations living on or near tribal lands. In addition, when considering policy initiatives to improve food environments so that all populations have access to healthy, affordable foods, Native American tribal lands should be prioritized. Additionally, tribal communities can use this information to identify barriers to the attainment of healthy food as a potential need to be addressed in their communities. This recognition can lead to innovative community-led solutions to prioritize healthy food access in underserved areas. Further research to better describe and understand the food environments surrounding tribal areas should include more precise measurements of food quality to capture intra-categorical differences of food outlets and a broadly representative qualitative sample to help contextualize the data.

## Supporting Information

S1 Dataset (CSV)

S1 Table SIC Codes and Their Corresponding Crude Categories (DOCX)

S1 Text Detailed Methods of Food Environment Geocoding and Classifying Food Venues (DOCX)

S2 Text Top Fast Food Restaurants included in the Fast Food Category by Text-Matching. (DOCX)
