# Supplementary material for: Food Environments around American Indian Reservations: A Mixed Methods Study
Source: PLoS One. 2016 Aug 25;11(8):e0161132. doi: 10.1371/journal.pone.0161132 (PMC4999270; doi:10.1371/journal.pone.0161132)
Supplement: S1 Table — (DOCX) [file pone.0161132.s002.docx]

**S1 Table. SIC Codes and Their Corresponding Crude Categories**

| SIC | SIC Title | Category |
| --- | --- | --- |
| 541101 | FOOD MARKETS | Supermarket |
| 541105 | GROCERS-RETAIL | Supermarket |
| 541107 | GROCERS-ETHNIC FOODS | Supermarket |
| 531110 | WHOLESALE CLUBS | Superstore |
| 543101 | FRUITS & VEGETABLES & PRODUCE-RETAIL | Produce markets |
| 543102 | FARM MARKETS | Produce markets |
| 543106 | PICK-YOUR-OWN FRUIT & VEGETABLES | Produce markets |
| 541108 | GROCERS-HEALTH FOODS | Healthy specialty stores |
| 542101 | SEAFOOD-RETAIL | Healthy specialty stores |
| 542105 | LOBSTERS | Healthy specialty stores |
| 542107 | MEAT-RETAIL | Healthy specialty stores |
| 542109 | SHRIMP-RETAIL | Healthy specialty stores |
| 542111 | MEAT MARKETS-KOSHER | Healthy specialty stores |
| 542116 | CRAB MEAT | Healthy specialty stores |
| 544102 | NUTS-EDIBLE | Healthy specialty stores |
| 549901 | HEALTH & DIET FOODS-RETAIL | Healthy specialty stores |
| 549907 | POULTRY-RETAIL | Healthy specialty stores |
| 549909 | FOODS-NATURAL | Healthy specialty stores |
| 549914 | EGGS-RETAIL | Healthy specialty stores |
| 549928 | DIETETIC FOOD PRODUCTS | Healthy specialty stores |
| 549932 | SOYFOODS | Healthy specialty stores |
| 549935 | ORGANIC FOODS & SERVICES | Healthy specialty stores |
| 541102 | SNACK PRODUCTS | Unhealthy specialty stores |
| 542106 | HAM SPECIALTY STORES | Unhealthy specialty stores |
| 542108 | SAUSAGES | Unhealthy specialty stores |
| 542110 | CAVIAR | Unhealthy specialty stores |
| 542113 | HAMS | Unhealthy specialty stores |
| 542114 | SMOKED FOODS | Unhealthy specialty stores |
| 542117 | MEATS-BARBECUED | Unhealthy specialty stores |
| 544101 | CANDY & CONFECTIONERY-RETAIL | Unhealthy specialty stores |
| 544103 | POPCORN & POPCORN SUPPLIES | Unhealthy specialty stores |
| 544105 | WEDDING CANDY & CONFECTIONERIES | Unhealthy specialty stores |
| 544106 | CANDY & COOKIE ARRANGEMENTS | Unhealthy specialty stores |
| 545102 | YOGURT | Unhealthy specialty stores |
| 545103 | CHEESE | Unhealthy specialty stores |
| 546101 | BAGELS | Unhealthy specialty stores |
| 546102 | BAKERS-RETAIL | Unhealthy specialty stores |
| 546103 | BAKERS-CAKE & PIE | Unhealthy specialty stores |
| 546104 | PIES | Unhealthy specialty stores |
| 546105 | DOUGHNUTS | Unhealthy specialty stores |
| 546107 | COOKIES & CRACKERS | Unhealthy specialty stores |
| 546108 | PRETZELS-RETAIL | Unhealthy specialty stores |
| 546109 | COOKIE SHOPS | Unhealthy specialty stores |
| 546111 | MATZOS | Unhealthy specialty stores |
| 549906 | SALT | Unhealthy specialty stores |
| 549924 | SEEDS-CHINESE PRESERVED | Unhealthy specialty stores |
| 549929 | COCONUT PRODUCTS | Unhealthy specialty stores |
| 581203 | ICE CREAM PARLORS | Unhealthy specialty stores |
| 581204 | WEDDING BAKERIES | Unhealthy specialty stores |
| 581217 | APPETIZERS & SNACKS ETC | Unhealthy specialty stores |
| 581218 | SODA FOUNTAIN SHOPS | Unhealthy specialty stores |
| 581221 | REFRESHMENT STANDS | Unhealthy specialty stores |
| 581229 | DELI-BAKERY | Unhealthy specialty stores |
| 581237 | DESSERT PREPARATIONS | Unhealthy specialty stores |
| 581241 | MILK BARS | Unhealthy specialty stores |
| 541104 | FOOD PRODUCTS-RETAIL | Mixed specialty stores |
| 542103 | FROZEN FOODS-RETAIL | Mixed specialty stores |
| 545101 | DAIRY PRODUCTS-RETAIL | Mixed specialty stores |
| 549910 | FOOD SPECIALTIES-RETAIL | Mixed specialty stores |
| 549912 | KOSHER FOODS | Mixed specialty stores |
| 549916 | ORIENTAL FOOD PRODUCTS | Mixed specialty stores |
| 549917 | CHINESE FOOD PRODUCTS | Mixed specialty stores |
| 549919 | JAPANESE FOOD PRODUCTS | Mixed specialty stores |
| 549920 | GOURMET SHOPS | Mixed specialty stores |
| 549921 | IMPORTED FOODS | Mixed specialty stores |
| 549923 | KOREAN FOODS | Mixed specialty stores |
| 549927 | MEXICAN & LATIN AMERICAN FOOD PRODUCTS | Mixed specialty stores |
| 549930 | BRITISH FOOD PRODUCTS | Mixed specialty stores |
| 581202 | GREEK FOOD PRODUCTS | Mixed specialty stores |
| 581227 | ITALIAN FOOD PRODUCTS | Mixed specialty stores |
| 549937 | VIETNAMESE FOODS | Full-service restaurants |
| 549941 | THAI FOOD | Full-service restaurants |
| 549946 | NATIVE AMERICAN FOODS | Full-service restaurants |
| 549947 | CARIBBEAN FOODS | Full-service restaurants |
| 581201 | LUAUS | Full-service restaurants |
| 581208 | RESTAURANTS | Full-service restaurants |
| 581224 | BARBECUE RESTAURANT | Full-service restaurants |
| 581226 | THEATRES-DINNER | Full-service restaurants |
| 581232 | CHOW MEIN & CHOP SUEY | Full-service restaurants |
| 581233 | JEWISH FOODS | Full-service restaurants |
| 581240 | CHUCK WAGON DINNERS | Full-service restaurants |
| 581244 | OYSTER BARS | Full-service restaurants |
| 581246 | CHILI PARLORS | Full-service restaurants |
| 581249 | RESTAURANTS-RESERVATIONS | Full-service restaurants |
| 581251 | RESTAURANTS-FAMILY DINING | Full-service restaurants |
| 533101 | VARIETY STORES | Convenience stores |
| 533102 | TRADING POSTS | Convenience stores |
| 539902 | COUNTRY STORES | Convenience stores |
| 541103 | CONVENIENCE STORES | Convenience stores |
| 549999 | MISCELLANEOUS FOOD STORES | Convenience stores |
| 581206 | FOODS-CARRY OUT | Carry-out restaurants |
| 581209 | DELICATESSENS | Carry-out restaurants |
| 581211 | CHICKEN DINNERS | Carry-out restaurants |
| 581213 | CAFETERIAS | Carry-out restaurants |
| 581214 | CAFES | Carry-out restaurants |
| 581219 | SANDWICHES | Carry-out restaurants |
| 581254 | RESTAURANTS-CYBER CAFES | Carry-out restaurants |
| 581205 | HAMBURGER & HOT DOG STANDS | Fast food |
| 581222 | PIZZA | Fast food |
| 581230 | RESTAURANTS-FOOD DELIVERY | Fast food |
| 581242 | MOBILE CONCESSIONS | Fast food |
