# Supplementary material for: Food Environments around American Indian Reservations: A Mixed Methods Study
Source: PLoS One. 2016 Aug 25;11(8):e0161132. doi: 10.1371/journal.pone.0161132 (PMC4999270; doi:10.1371/journal.pone.0161132)
Supplement: S1 Text — (DOCX) [file pone.0161132.s003.docx]

**S1 Text. Detailed Methods of Food Environment Geocoding and Classifying Food Venues**

Data on all food businesses in California in 2013 were obtained from InfoUSA. Classifying all food businesses involved multiple steps. First, we considered all businesses with SIC codes that start with 53, 54, 58 (which correspond to General Merchandise Stores, Food Stores, Eating & Drinking Places) to be potential food businesses. We classified each food business into a food venue type using the primary and secondary Standard Industrial Classification (SIC) codes. 111 different SIC codes could be used as a primary or secondary SIC to describe type of business for each food venue. We assigned all primary and secondary SIC codes to one of 14 crude categories of food venues (See Supplemental Appendix 2 for list of included SIC codes and their corresponding crude categories). If the primary and secondary SIC for one food business belonged to the same food venue category or if the food business had only one SIC code, the food business was classified by this single category. For food businesses whose primary and secondary SIC code belonged to two different crude categories, each unique combination of the two categories was individually evaluated by examining the food businesses with that combination and making a determination of the best single category for those food venues.

Special modification was then made to capture the top fast food chains in USA for the fast food category. We used an adapted list of the top 50 fast food chains (<http://www.qsrmagazine.com/reports/qsr50-2014-top-50-chart>), excluding those food venues that would belong to other categories according to the definitions used in our categorization (e.g. Jamba Juice) or those that did not exist in California (e.g. Tim Hortons) (See Supplemental Appendix 3 for list of included fast food restaurants). Therefore, the final fast food category contained both food businesses identified based on SIC codes (such as pizza and hamburgers venues) as well as venues from the adapted top 50 list. Each food business in the analysis belonged exclusively to one of the 14 categories.

All the relevant food businesses were then geocoded into point shapefiles using ArcGIS by either USA street address or USA point address. 114 out of 107,612 food addresses were manually matched if it couldn’t be automatically matched or if it was incorrectly matched. An additional 153 food businesses were mislocated beyond the scope of California due to incorrect addresses and therefore excluded from the analysis.
