# Supplementary material for: Food Environments around American Indian Reservations: A Mixed Methods Study
Source: PLoS One. 2016 Aug 25;11(8):e0161132. doi: 10.1371/journal.pone.0161132 (PMC4999270; doi:10.1371/journal.pone.0161132)
Supplement: S2 Text — (DOCX) [file pone.0161132.s004.docx]

**S2 Text. Top Fast Food Restaurants included in the Fast Food Category by Text-Matching.**

"Mc Donald's", "SUBWAY", "Wendy's", "Burger King", "Taco Bell", "Taco Bell/KFC", "Pizza Hut", "Chick-Fil-A", "KFC", "Panera Bread", "Sonic Drive-In", "Domino's Pizza", "Carl's Jr", "Hardee's", "Chipotle Mexican Grill", "Jack in the Box", "Arby's", "Little Caesars Pizza", "Papa John's Pizza", "Popeye's Chicken & Biscuits", "Panda Express", "Jimmy John's", "Five Guys Burgers & Fries", "Church's Chicken", "Papa Murphy's Take 'N' Bake", "Checkers Drive-In Restaurant", "Long John Silver's", "Del Taco", "El Pollo Loco", "Quiznos", "Boston Market", "Qdoba Mexican Grill", "Jason's Deli", "In-N-Out Burger", "Wingstop", "Baskin-Robbins", "Moe's Southwest Gril", "Dairy Queen", "Einstein Bros Bagels"
